# Spin-polarized current injection induced magnetic reconstruction at oxide interface

**DOI:** 10.1038/srep40048

**Published:** 2017-01-04

**Authors:** F. Fang, Y. W. Yin, Qi Li, G. Lüpke

**Affiliations:** 1 Department of Applied Science, College of William & Mary, Williamsburg, Virginia 23187, USA; 2 Department of Physics, Pennsylvania State University, University Park, Pennsylvania 16802, USA

## Abstract

Electrical manipulation of magnetism presents a promising way towards using the spin degree of freedom in very fast, low-power electronic devices. Though there has been tremendous progress in electrical control of magnetic properties using ferromagnetic (FM) nanostructures, an opportunity of manipulating antiferromagnetic (AFM) states should offer another route for creating a broad range of new enabling technologies. Here we selectively probe the interface magnetization of SrTiO_3_/La_0.5_Ca_0.5_MnO_3_/La_0.7_Sr_0.3_MnO_3_ heterojunctions and discover a new spin-polarized current injection induced interface magnetoelectric (ME) effect. The accumulation of majority spins at the interface causes a sudden, reversible transition of the spin alignment of interfacial Mn ions from AFM to FM exchange-coupled, while the injection of minority electron spins alters the interface magnetization from C-type to A-type AFM state. In contrast, the bulk magnetization remains unchanged. We attribute the current-induced interface ME effect to modulations of the strong double-exchange interaction between conducting electron spins and local magnetic moments. The effect is robust and may serve as a viable route for electronic and spintronic applications.

Magnetoelectric (ME) materials engineered for electrical control of magnetic properties have great potential for future development of low-power spintronics and magnetic random access memories^1^,^2^,^3^,^4^,^5^. In a more general sense, ME effects include not only the coupling between magnetic and electric order parameters^6^ but also involve related phenomena such as electrically controlled magneto-crystalline anisotropy^3^,^7^,^8^,^9^,^10^, exchange bias^11^,^12^ and spin transport^13^,^14^,^15^,^16^,^17^. Tailoring these phenomena in engineered thin-film heterostructures opens unexplored avenues for using the spin degree of freedom in electronic devices. For example, Yin *et al*.^18^ observed an enhanced tunneling electroresistance and significant manipulation of spin injection by inserting an ultrathin La_0.5_Ca_0.5_MnO_3_ (LCMO) barrier in the junction of La_0.7_Sr_0.3_MnO_3_/BaTiO_3_/La_0.7_Sr_0.3_MnO_3_. The results suggest a ferroelectrically induced metal-insulator phase transition in the LCMO layer that is of ME origin. In this study, we discover a new spin-polarized current injection induced ME effect that alters the interface magnetization of SrTiO_3_/La_0.5_Ca_0.5_MnO_3_/La_0.7_Sr_0.3_MnO_3_ (STO/LCMO/LSMO) heterojunction.

The STO/LSMO interface has been well studied^19^, because of its relevance in various electronic devices, such as diodes^20^,^21^, transistors^22^, and magnetic tunnel junctions^23^. Although working devices have been reported, it is well known that the properties of LSMO are deteriorating at the interface^24^, resulting in an interfacial “dead layer” for both conductivity and magnetization^25^. This reduction has been attributed to either a valence change at the polar interface^26^, a change in orbital ordering^27^, or interfacial strain^28^,^29^. A. Tebano *et al*.^27^ provided microscopic evidence that an orbital reconstruction exists at the interface in LSMO films, independent of the chemical nature of the substrate and the presence of capping layer, suggesting an intrinsic interfacial phenomenon. C. Aruta *et al*.^30^ demonstrated that an interfacial 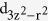 orbital occupation, in contrast to 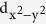 orbital occupation in LSMO bulk, favors the C-type antiferromagnetic (AFM) spin ordering. The easy axis of the C-AFM phase is oriented along the surface normal, which results in anisotropy of exchange coupling and spin hopping. Thus, careful studies to clarify the relationship between magnetic ordering and electronic properties at the STO/LSMO interface are of great interest.

## Results

**Figure 1:**
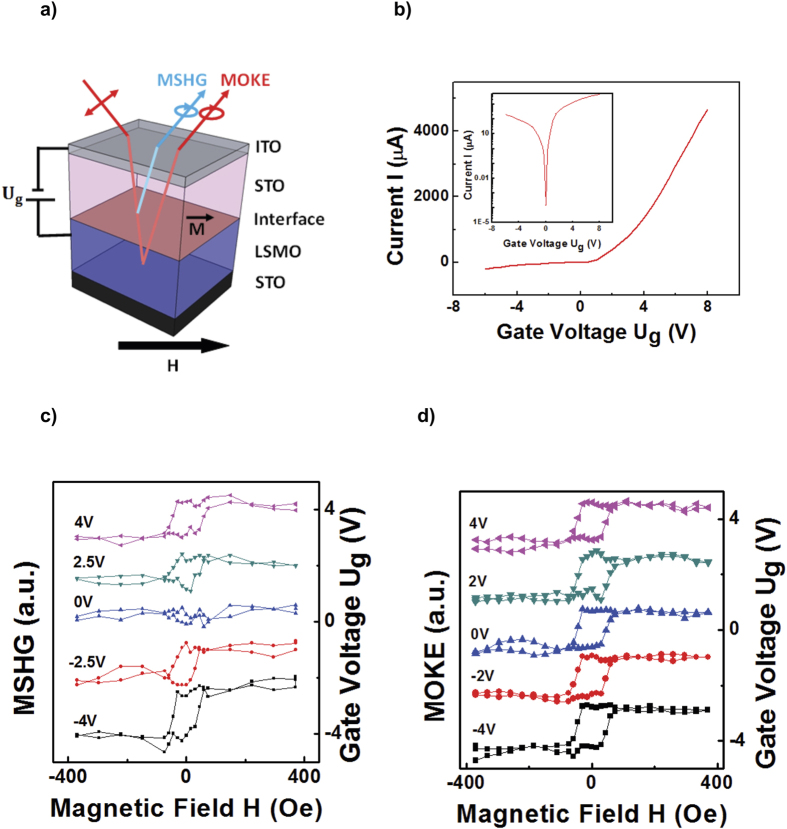
Measurement geometry, I–V data, MSHG, and MOKE loops from oxygen-rich STO/LCMO/LSMO heterostructure. (**a**) Schematic of the optical measurements. MOKE measures the bulk magnetization of the LSMO film, while MSHG selectively probes the interface magnetization only. The magnetic field *H* is applied in plane, i.e. in longitudinal MOKE/MSHG geometry. (**b**) I–V curve exhibiting diode effect. Magnetic hysteresis loops from (**c**) MSHG and (**d**) MOKE measurements as a function of gate voltage *U*_*g*_. The interfacial LSMO layer exhibits magnetic transitions, while the bulk LSMO maintains the FM state. All measurements are performed at 80 K.

In order to selectively probe the interface magnetization of STO/LSMO heterojunction, we use magnetization-induced second-harmonic generation (MSHG). A 1-nm thick La_0.5_Ca_0.5_MnO_3_ interlayer is inserted between LSMO and STO to improve the properties of the interfacial LSMO layer. Indium-tin-oxide (ITO) and LSMO layer serve as top and bottom electrodes to apply a gate voltage *U*_*g*_ (Fig. 1a). The ITO (30 nm)/STO (200 nm)/LCMO (1 nm)/LSMO (50 nm) heterostructures are epitaxially grown on STO (100) substrates by pulsed laser deposition (see Supplementary Information). STO/LCMO/LSMO heterostructures are processed at different growth conditions (see Supplementary Information), resulting in oxygen-rich and oxygen-poor STO layers. The sufficient native oxygen vacancies in STO are double shallow donors^31^, yielding electron concentrations of 5 × 10^17^/cm^3^ (2 × 10^18^/cm^3^) for the oxygen-rich (oxygen-poor) heterostructures, as determined from CV measurements. The I–V curves exhibit good diode characteristics with rectifying effect at negative voltage, as displayed for the oxygen-rich heterostructure in Fig. 1b. The MSHG technique is well suited for probing the interfacial magnetic state where the inversion symmetry is broken^32^,^33^,^34^. The characteristic length scale for our MSHG data is the length of one unit cell, because second-harmonic generation is forbidden (in dipole approximation) in centrosymmetric media. For comparison, magneto-optical Kerr effect (MOKE) measurements are carried out to detect the LSMO bulk magnetic properties (see Supplementary Information). All of the experiments are performed at 80 K, which is well below the Curie temperature of LSMO.

Figure 1c displays voltage-dependent MSHG loops for the oxygen-rich heterostructure varying from −4 V to +4 V. MSHG loops are observed at negative voltage but disappear near zero voltage. The loops reappear at +2.5 V. In contrast, MOKE loops are always observed over the whole voltage range (Fig. 1d). Since coercive fields of MSHG and MOKE loops are very similar, we attribute this phenomenon to a new interfacial ME effect in the LSMO layer. The magnetic properties of the ultrathin LCMO interlayer are not observed or distinguished, as it initially is AFM at zero gate voltage and can be tuned to other states by different carrier injection (a special kind of doping)^18^.

**Figure 2:**
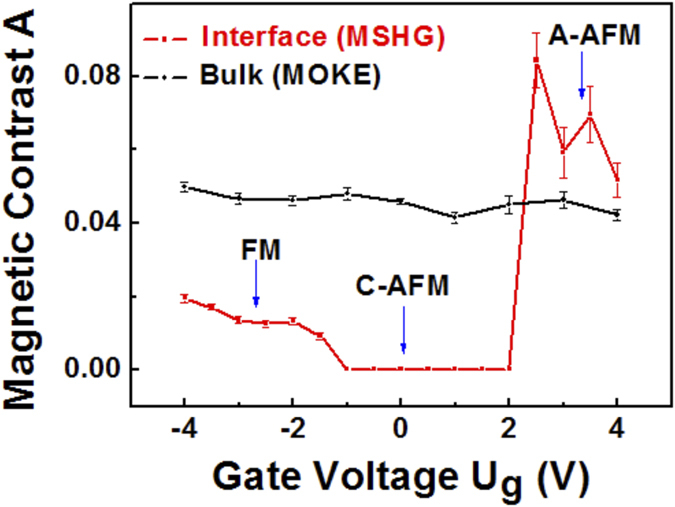
Magnetic contrast from oxygen-rich STO/LCMO/LSMO heterostructure. Magnetic contrast *A* for MSHG (red curve) and MOKE (black curve), determined from hysteresis loops as a function of gate voltage *U*_*g*_. The oxygen-rich BTO/LCMO/LSMO heterojunction exhibits interface magnetic transitions at *U*_*g*_ = −1 V (FM to C-AFM) and *U*_*g*_ = +2.5 V (C-AFM to A-AFM). All measurements are performed at 80 K.

To elucidate the observed phenomenon, we consider the magnetic contrast of the hysteresis loop, which is defined as:


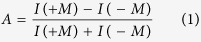


where *I(+M)* and *I(−M)* are the intensities for the two magnetizations (see Supplementary Information). The magnetic contrast obtained from MSHG and MOKE loops are displayed in Fig. 2, represented by red and black curves, respectively. The oxygen-rich STO/LCMO/LSMO heterojunction exhibits interface magnetic transitions at *U*_*g*_ = −1 V (FM to C-AFM) and *U*_*g*_ = + 2.5 V (C-AFM to A-AFM).

## Discussion

**Figure 3:**
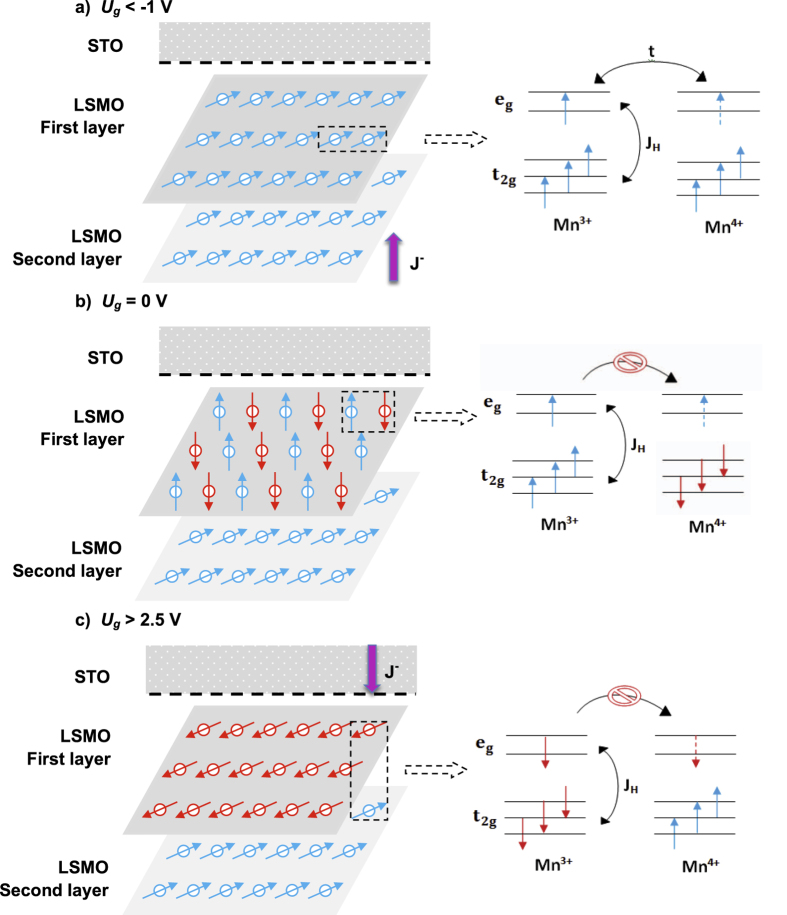
Model of spin alignment at STO/LSMO interface. (**a**) At reverse bias, majority spins (blue arrows) accumulate at the interface due to the electron current *J*^*−*^. The majority spins are double-exchange coupled (right panel), leading to a ferromagnetic state of interfacial LSMO. (**b**) At zero bias, the interfacial LSMO layer exhibits C-Type AFM phase due to orbital reconstruction. The spin-hopping process *t* is blocked by the strong interaction with the local spins due to the large Hund’s rule coupling *J*_*H*_ (right panel). (**c**) At forward bias, majority spins (blue arrows) flow across the LSMO layer by the spin-hopping process *t*. In contrast, minority spins (red arrows) accumulate at the interface, since the spin-hopping process *t* is blocked (right panel). The AFM super-exchange interaction of t_2g_ electrons between neighboring Mn ions dominates, and the interfacial LSMO layer assumes A-type AFM phase.

The spin alignment at interfacial LSMO layer, corresponding to the MSHG contrast, is depicted in Fig. 3. For simplicity, the LCMO interlayer is not shown. At reverse bias (*U*_*g*_ < −1 V), majority spin-up electrons will accumulate at the LSMO side driven by the spin-polarized electron current *J*^*−*^ generated in the half-metallic LSMO layer (Fig. 3a). The strong double-exchange coupling between the conducting e_g_ spin-up electrons and the local moments of t_2g_ electrons of Mn ions favors the FM configuration, as depicted on the right-hand-side of Fig. 3a. At zero bias voltage (Fig. 3b), the interfacial LSMO exhibits an insulating C-type AFM state because of interfacial 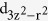 orbital occupation^30^. The 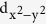 orbitals are unoccupied at the interface^27^, thus weakening the double-exchange mechanism due to less spin hopping, as depicted on the right-hand-side of Fig. 3b. The super-exchange interaction of t_2g_ electrons between neighboring Mn ions would then favor a C-AFM configuration near the interface. The spins are oriented preferentially along the surface normal (z direction). Thus, no magnetic contrast is observed in the longitudinal geometry used for the MSHG measurements.

When a forward bias voltage is applied, the most important process is the injection of minority spins from STO to LSMO near the interface (Fig. 3c). The majority spins flow across the LSMO layer by spin-hopping process *t*. In contrast, the minority spins will accumulate at the interface, since the spin-hopping process *t* is blocked by the strong interaction with the local spins due to the large Hund’s rule coupling *J*_*H*_, as depicted on the right-hand-side of Fig. 3c. Hence, the minority spins will accumulate in the first Mn layer near the interface. For *U*_*g*_ > +2.5 V, a transition to A-type AFM state occurs in the interfacial LSMO layer, since FM order is favored intralayer as the 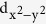 orbitals become occupied, while AFM coupling occurs interlayer. In contrast, the LSMO bulk maintains the FM configuration independent of applied bias due to the double-exchange interaction, as the minority spins are confined to the interface. A large MSHG contrast is observed with A-type AFM configuration (Fig. 2), because the easy-axis is oriented in plane^30^ and MSHG selectively probes the magnetization at the interface where the inversion symmetry is broken, i.e., the first unit-cell layer of LSMO. The magnetic reconstruction occurs at the interface and does not affect the bulk magnetic state. Hence the MOKE contrast does not change with gate voltage modulation (Fig. 2).

**Figure 4:**
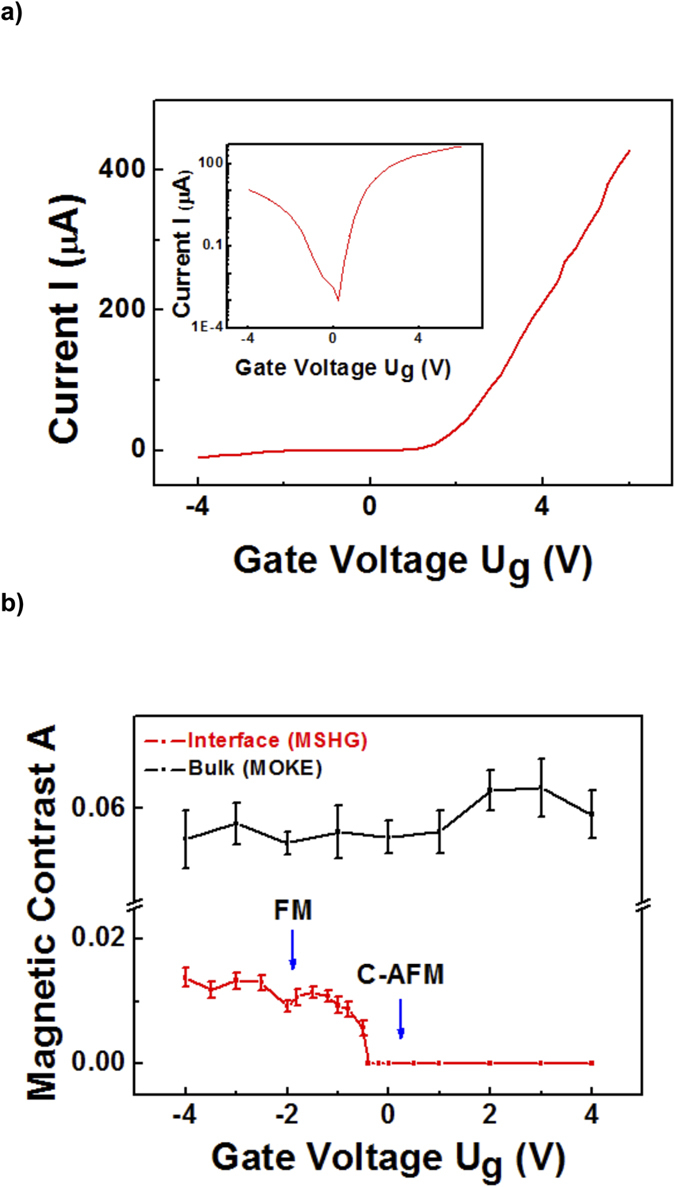
I–V data and magnetic contrast from oxygen-poor STO/LCMO/LSMO heterostructure. (**a**) I–V curve exhibiting diode effect. (**b**) Magnetic contrast *A* for MSHG (red curve) and MOKE (black curve), determined from hysteresis loops as a function of gate voltage *U*_*g*_. The oxygen-poor STO/LCMO/LSMO heterojunction exhibits an interface magnetic transition at *U*_*g*_ = −0.5 V (FM to C-AFM). All measurements are performed at 80 K.

Further insight into the observed interface ME phenomenon is provided from dopant dependent studies of the STO layer. Figure 4a displays the I–V curve of the oxygen-poor STO/LCMO/LSMO heterostructure exhibiting good diode characteristics. We note that the oxygen-poor STO layer is thicker (300 nm) than the oxygen-rich one as the thinner one is too leaky to afford high gate voltage. Voltage-dependent MSHG and MOKE loops are measured and the magnetic contrasts are displayed by red and black curves in Fig. 4b, respectively. An AFM-FM transition is observed at *U*_*g*_ = −0.5 V which is closer to zero bias than for the oxygen-rich heterostructure (−1V). The higher doping level of STO in the oxygen-poor heterostructure enhances the e_g_ electron concentration of the interfacial LSMO layer, pushing it closer to the FM phase. Hence a lower spin-polarized electron current *J*^*−*^ is required to induce the AFM-FM transition. On the other hand, the transition to A-type AFM phase is not observed at positive gate voltage, because the forward current is not large enough (<600 μA), i.e. the injected minority spins are not sufficient to drive the magnetic transition.

The observed interfacial magnetoelectric coupling mechanism is conceptually different from those known previously, such as ferroelectric polarization-induced changes in the lattice strain or nature of chemical bonding, and/or charge (carrier) modulation at the multiferroic heterojunction^15^. Both can affect the FM moments at the interface of LSMO layer, as expected from their critical phase-competitive nature in magnetism. We can exclude the conventional magneto-elastic strain effect, because the piezoelectric effect in STO is very small, d_31_~10^−13^ V/m, which is three orders of magnitude smaller than in PZT. We can also exclude the common magnetoelectric effect, because STO is an incipient ferroelectric. Furthermore, our dopant-dependent study shows that for higher electron concentration of STO a smaller negative bias is required to switch the magnetic order from AFM to FM type in the interfacial LSMO layer. This indicates that the magnetic transition is not driven by the electric field in the depletion layer, which is larger in the sample with the higher doping concentration requiring a more negative voltage to switch the magnetic order. Here, the accumulation of majority spins at the interface driven by a spin-polarized current causes a sudden, reversible transition of the spin alignment of interfacial Mn ions from AFM to FM exchange-coupled, while the injection of minority spins under forward bias alters the interface magnetization from C-type to A-type AFM state. The results are important for the transport properties of magnetic tunneling junctions, because an interfacial magnetic transition may notably change the spin polarization of the tunneling current and thus be decisive for tunneling magnetoresistance.

## Methods

### Sample fabrication and characterization

The oxide multilayers were epitaxially grown by pulsed laser deposition (KrF laser 248 nm) onto (001)-oriented SrTiO_3_ substrates. See Supplemental Information for a detailed description of the sample characterization.

### MOKE measurements

In the longitudinal MOKE studies, we measured the FM magnetization of LSMO layer by irradiating the sample with p-polarized light and detecting the s-component of the reflected light with a photodiode. The external magnetic field is applied in-plane along the LSMO [100] direction at 80 K. A detailed discussion of the magnetic properties of the samples is given in the Supplemental Information.

### MSHG measurements

MSHG experiments are performed with a Ti:sapphire amplifier system generating 200 femtosecond pulses with 4 μJ energy at 250 kHz repetition rate and 800 nm wavelength. The attenuated laser beam (50 mW) with S-polarization is focused to a 200 μm diameter spot on the sample at an angle of incidence of 40°. A small S-polarized MSHG signal (400 nm) is generated in the direction of the reflected laser beam, and is detected with a high signal-to-noise ratio photomultiplier. Proper filtering is required to separate the MSHG light from the fundamental laser beam. The external magnetic field is applied on the sample plane along [100] direction.

## Additional Information

**How to cite this article**: Fang, F. *et al*. Spin-polarized current injection induced magnetic reconstruction at oxide interface. *Sci. Rep.* **7**, 40048; doi: 10.1038/srep40048 (2017).

**Publisher's note:** Springer Nature remains neutral with regard to jurisdictional claims in published maps and institutional affiliations.

## Supplementary Material

Supplementary Information
