# Supplementary material for: Spin-polarized current injection induced magnetic reconstruction at oxide interface
Source: Sci Rep. 2017 Jan 4;7:40048. doi: 10.1038/srep40048 (PMC5209677; doi:10.1038/srep40048)
Supplement: Supplementary Information [file srep40048-s1.pdf]

Spin-polarized current injection induced magnetic reconstruction at oxide interface

F. Fang[1], Y. W. Yin[2], Qi Li[2], and G. Lüpke[1,*]

[1]*Department of Applied Science, College of William & Mary, Williamsburg, Virginia 23187, USA*

[2]*Department of Physics, Pennsylvania State University, University Park, Pennsylvania 16802, USA*

Supplementary Information

Sample preparation:

The ITO (30 nm)/STO (200 nm)/LCMO (1nm)/LSMO (50 nm) oxide heterostructures were grown on $SrTiO_3$ (001) substrates using a multi-target pulsed-laser deposition system (KrF excimer laser with λ = 248 nm). The epitaxial LSMO layer of 50 nm, a few unit cell LCMO layer, and STO layer of 200 nm were deposited *in situ* at a temperature of 750°C in flowing oxygen of 300 mtorr. The heterostructures were cooled down in either 500 torr oxygen pressure (oxygen-rich sample) or in 300 mtorr oxygen pressure (oxygen-deficient sample). The latter results in higher carrier concentration (n-type) than that of the oxygen-rich sample. The ITO layer is deposited in situ at 50°C in 10-mtorr oxygen.

The surface morphology of the LSMO and LCMO/LSMO films grown on STO substrates have been characterized by using atomic force microscopy (AFM) (Asylum Research-MFP-3D), which show atomically flat surface with the RMS roughness ~0.2-0.4 nm. Figure S1 shows a representative AFM image of an as-grown LSMO (50 nm) film (RMS roughness ~0.29 nm). Epitaxial LSMO and STO heterostructures made by pulsed-laser deposition have been reported by many groups in the past including our group.[1,2] Cross sectional TEM characterization of those samples have all shown atomically sharp interfaces. This work was conducted following our recent study on LSMO/BTO/LCMO/LSMO heterostructures made using similar conditions and reported previouly.[3] Cross-sectional annular dark-field (ADF) scanning transmission electron microscopy (STEM) image and the low-loss electron energy loss spectroscopy (EELS) mapping have been reported in Ref. 3 and a continuous LCMO layer with expected thickness and sharp interfaces have been obtained.

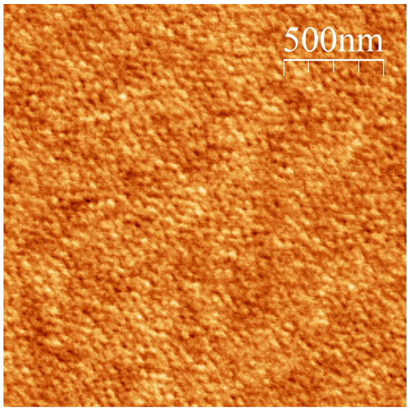

Figure S1. Morphological characterization of a LSMO(50nm) film (RMS roughness~0.29nm).

MSHG & MOKE:

MSHG experiments are performed with a Ti:sapphire amplifier system (Coherent) generating 200 femto-second pulses with 4 μJ energy at a center wavelength of 800 nm and a repetition rate of 250 kHz. The attenuated laser beam (~40mW) with S-polarization is focused to a 200-μm diameter spot on the sample at an angle of incidence of 40º. The S-polarized MSHG signal is generated in the direction of the reflected laser beam, and is detected with a high signal-to-noise ratio photomultiplier tube (PMT). Effective filtering and separation is required to separate the MSHG light from the fundamental laser beam. Magneto-Optical Kerr Effect (MOKE) experiments are carried out with similar configuration as MSHG, except that the laser beam with P-polarization is incident to the sample and the Kerr signal (800 nm) is detected with a high-speed silicon detector after an S-polarized analyzer.

Magnetic Contrast:

The magnetic contrast is defined by:

$$A = \frac{I(+M) - I(-M)}{I(+M) + I(-M)} \tag{1}$$

where *I(+M)* and *I(-M)* are the intensities for the two magnetizations (Fig. S2).

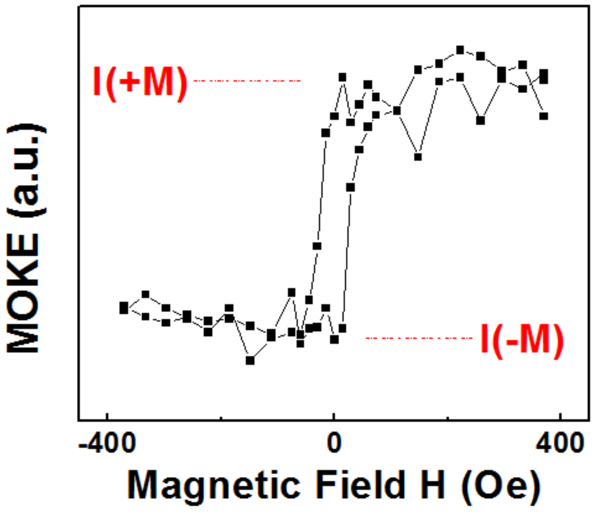

Figure S2: MOKE loop from oxygen-poor STO/LCMO/LSMO heterojunction
MOKE hysteresis loop indicating I(-M) and I(+M) used to determine the magnetic contrast *A* defined in Eq. (1). MOKE measurement is performed at 80 K.

1 Lu, Y. *et al.* Large magnetotunneling effect at low magnetic fields in micrometer-scale epitaxial La0.67Sr.033MnO3 tunnel junctions. *Phys. Rev. B* **54**, R8357-R8360 (1996).

2 Wertz, E. T. & Li, Q. Magnetoresistance after initial demagnetization in La[sub 0.67]Sr[sub 0.33]MnO[sub 3] / SrTiO[sub 3] / La[sub 0.67]Sr[sub 0.33]MnO[sub 3] magnetic tunnel junctions. *Appl. Phys. Lett.* **90**, 142506, doi:10.1063/1.2718481 (2007).

3 Yin, Y. W. *et al.* Enhanced tunnelling electroresistance effect due to a ferroelectrically induced phase transition at a magnetic complex oxide interface. *Nature Mater.* **12**, 397-402, doi:10.1038/nmat3564 (2013).
